# Phenotypic and Genomic Insights Into *Bacillus* spp. and *Peribacillus* spp. of Spanish Olive Groves With Biotechnological Potential

**DOI:** 10.1111/1758-2229.70053

**Published:** 2024-11-27

**Authors:** Julia Manetsberger, Natacha Caballero Gómez, Nabil Benomar, Graham Christie, Hikmate Abriouel

**Affiliations:** ^1^ Area of Microbiology, Department of Health Sciences, Faculty of Experimental Sciences University of Jaén Jaén Spain; ^2^ Department of Chemical Engineering and Biotechnology University of Cambridge Cambridge UK

**Keywords:** *Bacillus* spp., biofilm, exosporium, olive sporobiota, spore resistance

## Abstract

Spore‐forming organisms are an integral component of the rhizosphere, providing plants with significant advantages. Previous studies determined the antimicrobial activity of the olive sporobiota, identifying five candidates of particular relevance, belonging to the *Bacillus subtilis* , *Peribacillus simplex* and *Bacillus cereus* clade. This study aimed to determine their biotechnological properties, safety aspects, spore structure and resistance, and interaction with the environment through a combined microbiological and genomic approach. We report on the ability of these strains to produce hydrolytic and surface‐active enzymes and provide evidence for differences in population behaviour through the formation of strong sessile or floating biofilms. Electron microscopic analysis revealed for the first time the presence of an exosporium layer in olive sporobiota isolates belonging to the *P. simplex* and *B. cereus* clade, including numerous pili‐like structures on the latter. Spores showed significant differences in their resistance to wet heat, oxidising agents, and UV exposure. Whole genome sequencing of isolate *Peribacillus frigoritolerans* yielded information on its antimicrobial compound biosynthesis and environmental safety. Overall, our findings provide insights into the phenotypic, morphological and genetic variations of spore‐formers from Spanish olive groves, which can be useful for the development of bioactive compounds in sustainable agriculture.

## Graphical abstract

Spore‐forming organisms isolated from Spanish olive groves produce hydrolytic and surface‐active enzymes. Population behaviour is influenced by the formation of strong floating and sessile biofilms. Whole genome sequencing confirms bacteriocin production and environmental safety for the *Peribacillus frigoritolerans* strain UJA_MA_369.

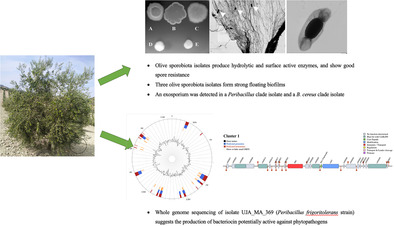

## 1 Introduction

Members of the *Firmicutes* family are ubiquitously present in nature and represent one of the predominant microorganisms in the plant rhizosphere (Radhakrishnan, Hashem, and Abd Allah 2017). These bacteria can provide plants with a variety of important competitive advantages, such as promoting plant growth, inducing systemic resistance and supporting the plants defence against pathogenetic attacks as well as increasing nutrient availability and nitrogen (N_2_) fixation amongst others (Manetsberger, Caballero Gómez, Soria‐Rodríguez, et al. 2023; Tsotetsi et al. 2022).

In addition to their beneficial properties for plants, their most distinguished feature is the ability to form endospores to withstand adverse environmental conditions and ensure the genus' survival, where others will surrender (Setlow 2006). The spores can resist harsh conditions, such as UV radiation, desiccation and pH fluctuations, making them highly interesting candidates for agricultural settings (Nicholson 2002; Setlow 2006). On the other hand, this resistance and persistence also make them a great challenge to clinical environments as well as food safety and quality as they stubbornly resist high temperature, sterilisation processes and harsh cleaning agents (Zegeye et al. 2021). This situation is aggravated by the bacteria's ability to tightly adhere to organic, inorganic and biological surfaces, both in the vegetative state (in form of biofilms) and as inert spores (Hayrapetyan et al. 2015; Klavenes et al. 2002; Ramarao and Lereclus 2006).

Essentially, all spores share the same general morphological features, which include the dehydrated spore core, the inner spore membrane and germ wall, the cortex and outer spore membrane and the spore coat, which have been extensively reviewed elsewhere (Driks 2009; McKenney, Driks, and Eichenberger 2013; McKenney and Eichenberger 2012; Plomp et al. 2014). The most important variance is the presence/absence of the loosely associated exosporium structure, present only in several species, such as the *Bacillus cereus* *sensu lato* (s.l.) group (Ball et al. 2008). The exosporium is directly implicated in the mechanical interaction of the spore with its immediate environment, such as surface adherence, spore aggregation, biofilm formation and virulence (Zegeye et al. 2021).

Recently, we identified the spore‐forming collective of Spanish olive groves—termed the culturable olive sporobiota—and demonstrated that these bacteria can largely resist environmental challenges often encountered in agricultural soils, such as heavy metals, fertilisers and common antibiotics (Manetsberger, Caballero Gómez, Benomar, et al. 2023).

Furthermore, five isolates of this cohort belonging to the *Bacillus subtilis* , *Peribacillus simplex* and *B. cereus* clade are natural antagonists against several important human and plant pests (Manetsberger et al. 2024). Notably, isolates as well as their cell‐free culture extracts are growth inhibitors of the bacterium *Xylella fastidiosa* and the fungus *Verticillium dahliae* (Manetsberger et al. 2024), which represent a global threat to many commercially relevant crops, including olive, almond and citric fruits (Delbianco et al. 2022; Sicard et al. 2018; Varo, Raya‐Ortega, and Trapero 2016). The identified olive sporobiota isolates could provide competitive advantages in the fight against these detrimental phytopathogens without disturbing the environment through addition of alien (chemical) substances, making them highly interesting candidates for further studies and development into biopesticides (Manetsberger, Caballero Gómez, Benomar, et al. 2023; Manetsberger et al. 2024).

Hence, the purpose of this study was to further examine the biotechnological properties of these strains, including the characterisation of environmental behaviour and interaction, spore structure and resistance as well as safety aspects through a combined microbiological and genomic approach. Strain UJA_MA_369 was chosen for in‐depth genomic analysis based on its antimicrobial potential as determined in our previous studies (Manetsberger, Caballero Gómez, Benomar, et al. 2023; Manetsberger et al. 2024) as well as a good combination of salt tolerance, resistance and sensitivity of spores towards different treatment as determined in this study.

## 2 Materials and Methods

### 2.1 Bacterial Strains and Growth Conditions

Bacterial strains used in this study are listed in Table 1. These strains are derived from the culturable olive sporobiota, which were previously identified and partially characterised by our group (Manetsberger, Caballero Gómez, Benomar, et al. 2023; Manetsberger et al. 2024). All strains were cultured routinely at 37°C in Lysogeny broth (LB) medium, if not stated differently. Spores were obtained via nutrient exhaustion by culturing cells on 2 × SG medium at 37°C for 3–5 days. Spores were harvested and purified by flooding the plates with ice‐cold sterile milli‐Q water, centrifugation and subsequent washing steps with ice‐cold water. Bacterial strains were stored in 25% glycerol at −20°C to −80°C until required, whereas spores were kept in sterile water at 4°C.

**TABLE 1:** Culturable olive sporobiota strains analysed in this study previously isolated and identified (Manetsberger, Caballero Gómez, Benomar, et al. 2023; Manetsberger et al. 2024).

Strain code used in this study	Olive sporobiota isolate	Relative location^a^	Clade identification	Strain designation by MALDI‐TOF
A	UJA_LIN_009	Soil	*Bacillus subtilis*	*Bacillus velezensis* CECT 30858
B	UJA_LIN_124	Endo	*Bacillus subtilis*	*Bacillus subtilis* CECT 30859
C	UJA_LIN_129	Endo	*Bacillus subtilis*	*Bacillus mojavensis* CECT 30857
D	UJA_MA_369	Soil	*Peribacillus simplex*	*Peribacillus simplex* CECT 30856
E	UJA_MA_406	Soil	*Bacillus cereus*	*Bacillus mycoides* CECT 30855

^a^ The relative location of isolation is specified, that is, from soil and leaf internal (endo).

### 2.2 Hydrolytic Enzyme Production

Olive sporobiota isolates were assessed regarding the production of environmentally and biotechnologically relevant enzymes (amylase, protease, cellulase and urease) as described previously (Afordoanyi et al. 2023). A total of 5 × 10 μL of cell culture, grown overnight, were distributed on basal medium agar (Afordoanyi et al. 2023) supplemented with either 1% skim milk powder (protease assay), 1% carboxymethyl cellulose (cellulase assay) or 1% starch (amylase). After 2 days incubation at 37°C, amylase plates were stained with iodine solution, whereas cellulose plates were stained for 1 h with 0.1% Congo Red followed by destaining with 1 M NaCl. The appearance of a halo or clearance zones (protease assay) around the bacterial spots was considered as enzymatic activities. Urease plates were prepared following the manufacturer's instructions and inoculated as described above. Colour change from rosé to pink indicated urease production.

### 2.3 Biofilm Formation

A fresh overnight culture was diluted (1:100) in LB, tryptic soy broth (TSB) or Muller Hinton broth (MHB), incubated at 37°C for 48 h (without agitation) and biofilm formation was assessed qualitatively.

Quantitative assessment of biofilm formation was performed as described previously (Caballero Gómez et al. 2022) with some modifications. A fresh overnight culture of the olive sporobiota strain was diluted 1:100 in LB on a 12‐well polystyrene microtiter plate yielding a final volume of 2 mL per well and incubated at 37°C. LB without bacteria was used as negative control. After 48 h incubation, the culture medium was carefully removed using a micropipette, allowing the floating biofilm to attach to the well bottom. Subsequent washing steps were omitted to avoid the loss of biofilm. Two millilitres of methanol was used to fix the bacteria to the well plate for 15 min, followed by airdrying of the wells. Biofilms were stained for 5 min at room temperature with 2% (V/V) crystal violet. Excess stain was removed carefully using a micropipette and wells were washed once with sterile MQ water. Addition of 33% (V/V) glacial acetic acid released the stain, and optical density was recorded at 620 nm. All experiments were performed in triplicate.

The cut‐off value (ODC) was set at three standard deviations (SDs) above the absorbance of the negative control (ODnc): ODC = ODnc + 3 × SD following previous methods (Fessia et al. 2022). Biofilm formation was hence classified as none (OD ≤ ODC), weak (ODC < OD ≤ 2 × ODC), moderate (2 × ODC < OD ≤ 4 × ODC) and strong (4 × ODC < OD). The following values were determined: ODC = 0.08, 2 × ODC = 0.16, 4 × ODC = 0.32.

### 2.4 Biosurfactant Production

To assess biosurfactant production in olive sporobiota isolates, an overnight culture was centrifuged at 14000 rpm for 10 min. Fifty microliters of the cell‐free supernatant was placed on parafilm and changes in surface tension were evaluated. Sterile Milli‐Q water and 10% sodium dodecyl sulphate (SDS) solution were used as control.

### 2.5 Tolerance to Saline Stress Conditions

Overnight cultures of olive sporobiota isolates were diluted (1:100) and inoculated in triplicate in 0.5 × LB supplemented with increasing NaCl concentrations (1%, 3%, 4.5%, 6% and 9% added salt). 0.5 × LB or inoculated 1× LB served as negative and positive control, respectively. The 96‐well microtiter plates were incubated at 37°C ± 0.3°C and analysed. Bacterial growth was monitored during 24 h, measuring the optical density at 595 nm for each well using a Tecan Infinite M200 multimode microplate reader equipped with monochromator optics. Before each measurement, orbital shaking conditions were selected (4 mm amplitude and 15 s shaking cycles). Measurements were taken every 60 min using the multiple‐reads‐per‐well mode (filled‐circle alignment, 3 × 3 spots, five reads per well, border 2000 mm).

### 2.6 Resistance Properties of Spores of Olive Sporobiota Isolates

Spore resistance properties were tested as described previously (Harwood and Cutting 1990) with slight modifications. All spore solutions were assayed at OD_600_ = 1 and experiments performed in triplicates.

#### 2.6.1 Temperature Resistance

Hundred microliters of OD_600_ = 1 spores in sterile water were incubated at 80°C for 0, 10, 30 and 60 min. Serial dilutions of the thermally treated spore suspensions were prepared in sterile water and 10 μL aliquots dispensed on LB agar medium. Plates were tilted slightly to allow the extension along the plate surface. Colony growth was assessed after overnight incubation at 37°C and colony forming units (CFUs) were determined.

#### 2.6.2 UV Resistance

Resistance to ultraviolet light was assessed at 254 nm (Jalali, Maghsoudi, and Noroozian 2020; Moeller et al. 2005). One millilitre of a spore solution (OD_600_ = 1) was exposed to UV radiation in an open sterile petri dish. Aliquots were removed at different time intervals and survival rate was assessed by plating 10 μL of serial dilutions on LB agar followed by 24 h incubation at 37°C.

#### 2.6.3 Resistance to Oxidising Agents/Disinfectants

The spores' resistance to hypochlorite was evaluated as described by Young and Setlow (2003) with slight modifications. Sodium hypochlorite (household bleach) was added to 1 mL of OD_600_ = 1 spores in sterile water at varying concentrations (1%, 5% and 10%) and incubated at room temperature for 4 h. Spores were removed from the solution by centrifugation at 14000 rpm for 5 min and resuspended in sterile water. Ten microliters of serial dilutions in sterile water were plated on LB agar and evaluated after overnight incubation at 37°C.

### 2.7 Electron Microscopy

Negative stain electron microscopy was used to visualize the spore ultrastructure. To do so, carbon formvar grids were placed on a drop of spore suspension for 1 min, followed by removing excess liquid through blotting with filter paper and staining on a drop of UranyLess EM stain (EMS) for 1 min. Transmission electron microscopy (TEM) images of spores were acquired using a JEOL JEM‐1010 transmission electron microscope (accelerating voltage of 80 kV) coupled with a Gatan 782 CCD camera. Scanning transmission electron microscopy (STEM) was performed on a Carl Zeiss MERLIN High Resolution Scanning Electron Microscope (FESEM) operated at 20 kV.

### 2.8 DNA Extraction, Genome Sequencing and Bioinformatic Characterisation

Genomic DNA was extracted from olive sporobiota bacterial cells, after overnight growth in LB liquid medium at 37°C using the Qiagen MagAttract HMW DNA Kit following the manufacturer's instructions. DNA quality and concentration were assessed using Nanodrop 2000, Fluorimetry with Qubit and the TapeStation 4200 bioanalyser. Genome sequencing, assembly and annotation experiments were performed at Biopolis (Valencia, Spain). The complete genome sequence for UJA_MA_369 was deposited at the EMBL Nucleotide Sequence Database (accession number PRJEB80151).

Two sequencing and hybrid assembly techniques were used to generate the closed genomes. Starting from 1 ng, the Illumina Nextera XT genotypes were sequenced in the Miseq equipment in 300 × 2 configuration. From 500 ng onwards, the long‐read library, Ligation sequencing gDNA‐Native barcoding was performed and sequenced in the PromethION 2 SOLO equipment in the R10.4.1 flowcell. FASTA files were obtained using these sequences and the Unicycler V2 software (Wick et al. 2017). The Proksee tool (Grant et al. 2023) was used for characterisation and visualisation of the bacterial genome.

The JSpeciesWS TCS and ANIb tool (Richter et al. 2016) was used for taxonomic identification, whereas open reading frame (ORF) prediction and annotation were performed using the NCBI's Prokaryotic Genome Annotation Pipeline (PGAP) (Tatusova et al. 2016). The BLASTp algorithm (Altschul et al. 1990) v2.12.0+ and the Comprehensive Antibiotic Resistance Database (CARD) v3.2.9 (Alcock et al. 2023) or Virulence Factors of Pathogenic Bacteria database (VFDB) were used to assess the presence of antibiotic resistance genes or virulence factors, respectively.

The BAGEL4 tool (Van Heel et al. 2018) was used to search for bacteriocins, whereas for the detection of biogenic amine production‐associated protein sequences, a BLASTp was performed against the database of biogenic amine production‐associated proteins (Guarcello et al. 2016) of UniProt. The online tool IslandViewer v4 (Bertelli et al. 2017) was used to detect genomic islands.

### 2.9 Statistical Analysis and Image Preparation

Images were analysed and assembled using the open‐source raster graphics editor GNU Image Manipulation Program (GIMP) (available at https://www.gimp.org/). If necessary, contrast was enhanced for better visualisation.

Mean values and SDs were calculated in Microsoft Excel 2019, and all analyses were performed in triplicate (biological and technical replicates). Datasets were compared using the one‐way analysis of variance (ANOVA) and Tukey's test. *p < 0.05 was considered significantly different.

## 3 Results

### 3.1 Assessment of Agriculturally Relevant Characteristics

The ability to produce biotechnologically relevant enzymes was assessed in olive sporobiota isolates (Table 2). The presence of proteolytic, cellulolytic, ureolytic and amylolytic enzymes was tested on minimal medium supplemented with the respective substrates. UJA_LIN_009 showed activity for all tested enzymes, namely cellulase, amylase, urease and protease. UJA_LIN_124, UJA_LIN_129 and UJA_MA_406 showed hydrolytic activity for three out of the four tested enzymes, devoid of one activity each (amylase, cellulase and amylase, respectively). UJA_MA_369 on the other hand showed the presence of only proteolytic and cellulolytic enzymes.

**TABLE 2:** Hydrolytic enzyme production of environmental relevance of selected olive sporobiota strains.

Strain	Cellulase	Amylase	Urease	Protease
UJA_LIN_009	+	+	+	+
UJA_LIN_124	+	−	+	+
UJA_LIN_129	−	+	+	+
UJA_MA_369	+	−	−	+
UJA_MA_406	+	−	+	+

*Note:* (+) indicates enzyme production as observed by the formation of a halo or clearance zones around the bacterial colonies after incubation; (−) no changes detected.

The strains were furthermore assessed for their ability to produce surface‐active molecules (Figure S1). UJA_MA_369 and UJA_MA_406 cell‐free culture medium displayed no changes in surface tension as compared to the negative control (sterile MQ water). The remaining three strains—UJA_LIN_009, UJA_LIN_124 and UJA_LIN_129—evidenced changes in surface tension, suggesting the production of surface‐active molecules (surfactins) and their secretion into the culture medium.

### 3.2 Biofilm Formation

**FIGURE 1:**
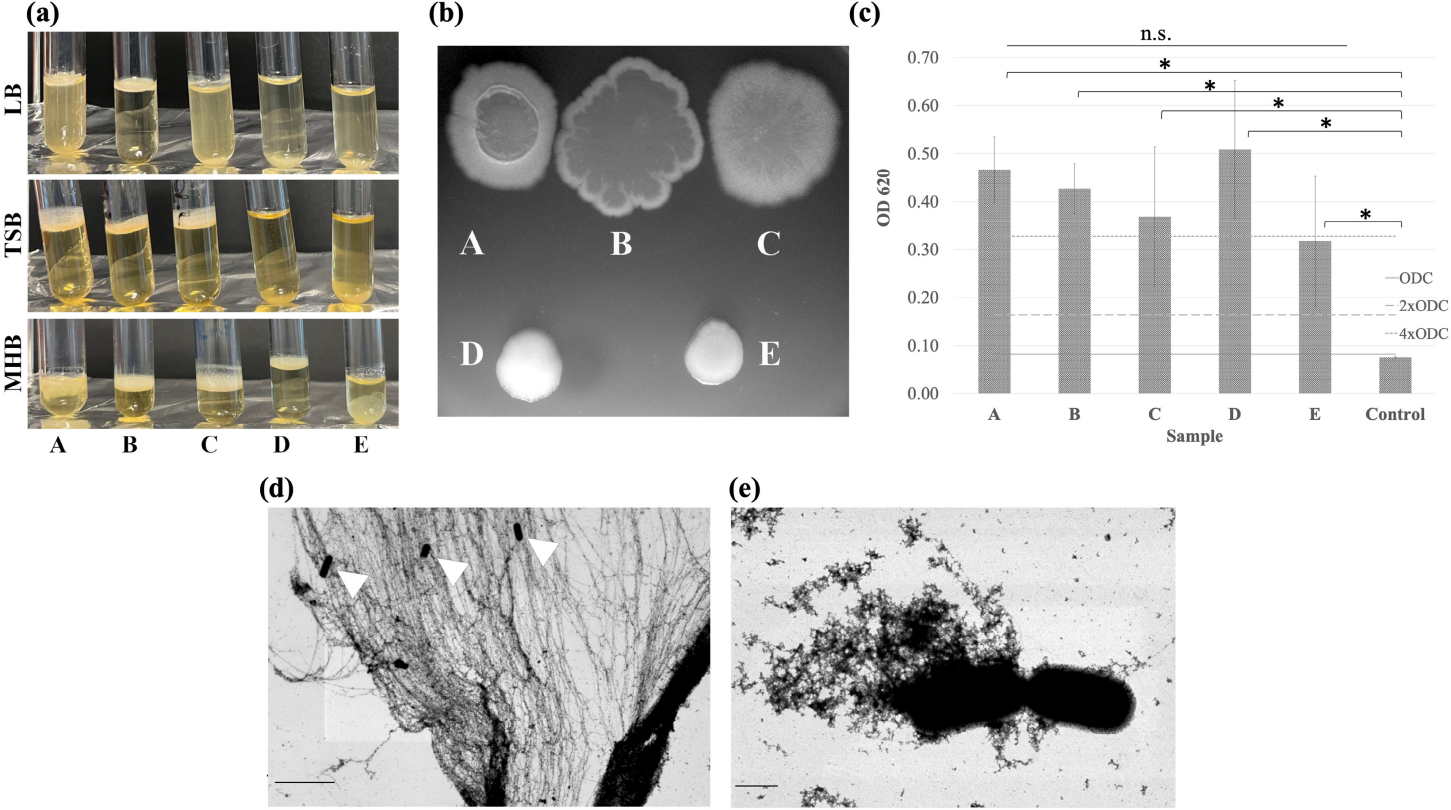
Evaluation of biofilm formation of selected olive sporobiota strains: (A) UJA_LIN_009, (B) UJA_LIN_124, (C) UJA_LIN_129, (D) UJA_MA_369 and (E) UJA_MA_406. (a) The effect of culture medium on floating/sessile biofilm formation is illustrated. Bacterial strains were incubated at 37°C overnight in Lysogeny broth (LB), tryptic soy broth (TSB) and Mueller Hinton broth (MHB). Floating biofilms are visible as layers on all broth surfaces (A, B), while bacterial pellet is visible for sample E in LB and TSB. Sample D showed biofilm formation only in MHB. (b) Colony morphology after overnight growth on LB agar at 37°C. (c) Quantification of biofilm formation for strains A‐E in LB medium measured through crystal violet assay compared to the negative control (Control). Thresholds for biofilm formation was classified as none (OD ≤ ODC), weak (ODC < OD ≤ 2ODC), moderate (2ODC < OD ≤ 4ODC), and strong (4ODC < OD). Results were analysed by one‐way‐ANOVA and Tukey test (*p* < 0.05). (*) indicates significant difference with the control; (n.s.) indicates no significant difference. (d) Representative scanning electron microscopy images of biofilms formed by vegetative cells of olive sporobiota isolates UJA_LIN_124 (size bar corresponds to 10 μm), and (e) UJA_MA_406 (size bar corresponds to 1 μm). White arrowheads indicate bacterial cells embedded within biofilm.

Growth of olive sporobiota isolates in liquid media in standing culture showed the formation of floating biofilms (pellicles) on the air/liquid interface, which was dependent on the culture medium (Figure 1a). Growth in Mueller Hinton broth (MHB) lead to the formation of floating biofilms for all isolates, with nearly clear culture medium, whereas growth in tryptic soy broth (TSB) lead to pellicle formation for UJA_LIN_009, UJA_LIN_124 and UJA_LIN_129, leaving the medium nearly clear, whereas UJA_MA_369 and UJA_MA_406 formed bacterial pellets. Stationary incubation in LB also showed pellicle formation for the first three strains; however, some turbidity was detected in the culture medium, indicating a mix of biofilm and planktonic cells for UJA_LIN_009 and UJA_LIN_129 (Figure 1a). Colony morphology did show similar trends (Figure 1b). Although showing slight differences in strains UJA_LIN_009, UJA_LIN_124 and UJA_LIN_129 formed colonies with mucous centres and fuzzy edges that quickly extended to cover the entire plate during longer incubation times. In contrast, UJA_MA_369 and UJA_MA_406 formed round, flat colonies with a shiny surface but lacking any extensions.

We then quantified biofilm formation of the olive sporobiota isolates. For those strains generating floating biofilms, culture medium was carefully removed from the culture plate, leaving the film to attach to the well bottom. Assays showed that all five tested strains generated biofilms in liquid LB cultures, with isolates UJA_LIN_009 (OD_620_ = 0.47 ± 0.07), UJA_LIN_124 (OD_620_ = 0.43 ± 0.05) and UJA_MA_369 (OD_620_ = 0.51 ± 0.14) characterised as strong biofilm producers (OD_620_ > 4 × ODC), and UJA_LIN_129 (OD_620_ = 0.0.37 ± 0.15) and UJA_MA_406 (OD_620_ = 0.31 ± 0.14) characterised as moderate‐to‐strong biofilm producers (see Section 2.3) (Figure 1c). Large variations in measurements could be attributed to the formation of stable surface‐associated floating biofilms and ensuing difficulties in the quantification. Another explanation could be the formation of submerged biofilms at the interface between the surface and the liquid. These can have a thick morphology and show large structural variations depending on the *Bacillus* strain (Bridier et al. 2011). However, they still remained within the characterisation range of biofilm strength.

Electron microscopic imaging of pellicles formed by UJA_LIN_129 as a representative of a pellicle‐producing strain showed the formation of sheets and filaments creating a large network of floating biofilms into which bacterial cells were tightly embedded (Figure 1d). In comparison, STEM images of UJA_MA_406 (no floating biofilm formation) showed vegetative cells floating freely in the growth medium. However, the strain also produced extracellular polymeric substances of granular appearance associated with the cells (Figure 1e).

### 3.3 Stress Tolerance to Saline Conditions

**FIGURE 2:**
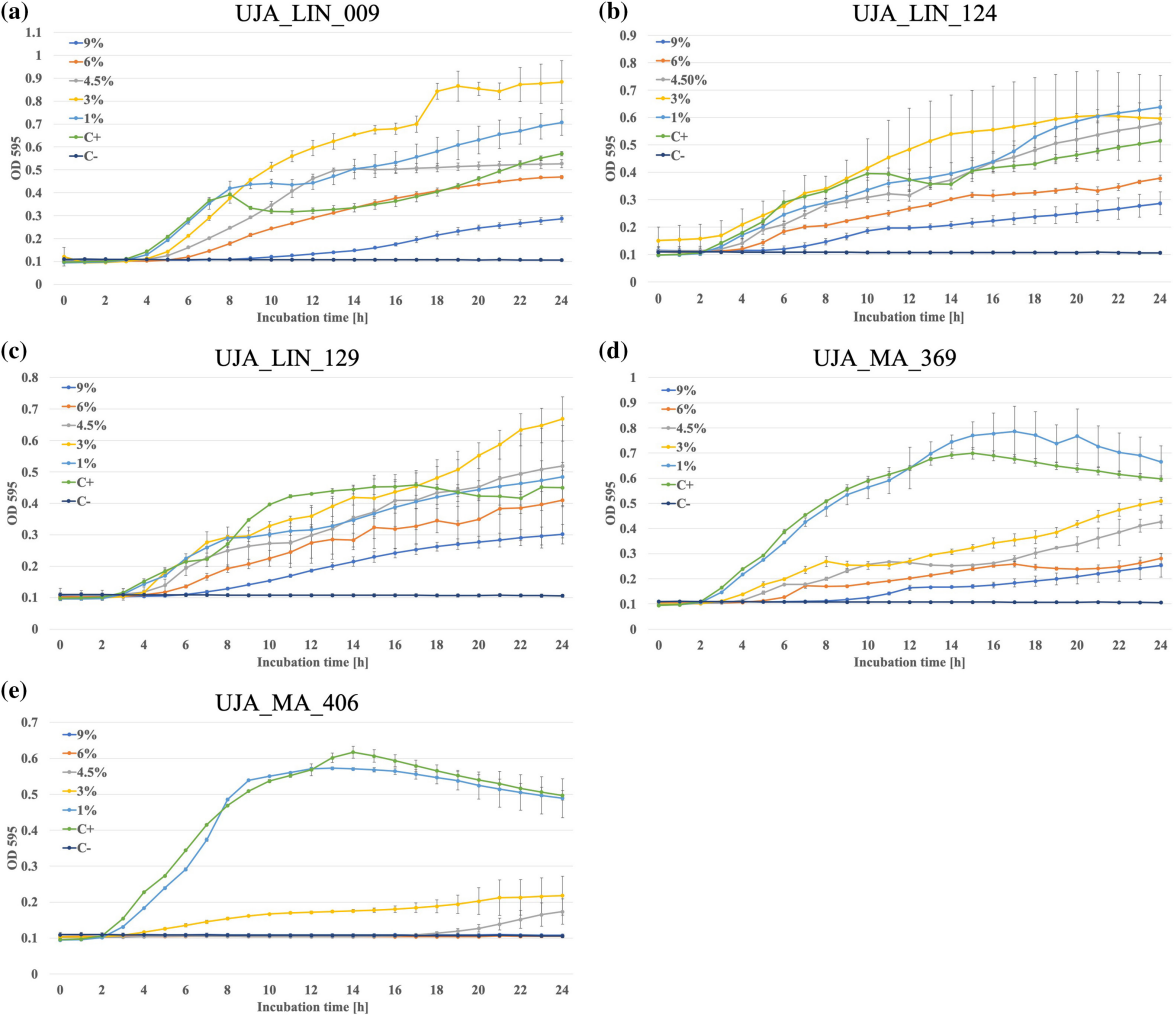
Bacterial growth curve to evaluate tolerance to different saline levels (1%–9%). (a) UJA_LIN_009, (b) UJA_LIN_124, (c) UJA_LIN_129, (d) UJA_MA_369 and (e) UJA_MA_406. Inoculated LB medium served as positive control (C+), and sterile 0.5× LB as negative control (C−).

The ability of the olive sporobiota isolates to tolerate and proliferate under different salt concentrations was assessed during 24 h when exposed to increasing saline concentrations (1%–9% added to 0.5× LB). Analysis showed large variations, both between strains and depending on salt concentration (Figure 2). Although showing variations in growth rate and growth potential, UJA_LIN_009 was able to grow both under low and high saline conditions. A 9% salinity slowed down bacterial growth and the isolate entered into exponential phase only after 16 h incubation, whereas 1%, 3%, 4% and 5% NaCl were well tolerated by the strains and yielded slightly increased bacterial growth as compared to the positive control. At 6% salt, it took the strain longer to adapt to the conditions; however, growth was then comparable to the control (Figure 2a). UJA_LIN_124 showed similar behaviour as the previous strain. All salt concentrations were tolerated at different levels. Salt concentration > 6% NaCl decelerated bacterial growth, whereas concentrations ranging at 1% or 4.5% were similar to the positive control. The 3% NaCl appeared to provide optimum conditions for bacterial growth, with a strong exponential phase from 3 to 18 h of incubation (Figure 2b). Measurements of optical density for UJA_LIN_129 showed large SDs, which are probably caused by strong floating biofilm formation in liquid culture. That said, a similar growth pattern as for UJA_LIN_124 could be observed, with good growth under low to medium saline conditions (1%–4.5% NaCl), whereas higher concentrations (> 6% NaCl) slightly inhibited bacterial growth (Figure 2c). On the contrary, UJA_MA_369 (Figure 2d) and UJA_MA_406 (Figure 2e) demonstrated lower tolerance to saline conditions than other isolates. Bacteria grown in nutrient broth supplemented with 1% NaCl showed a typical growth curve with exponential phase until 9–14 h of incubation before entering into stationary and decreased cell density at 18–20 h of incubation which probably indicated spore release. Salinity > 3% on the other hand progressively inhibited bacterial growth with increasing salt concentration (UJA_MA_369) or completely inhibited growth for concentrations > 6% (UJA_MA_406).

### 3.4 Spore Resistance Properties

**FIGURE 3:**
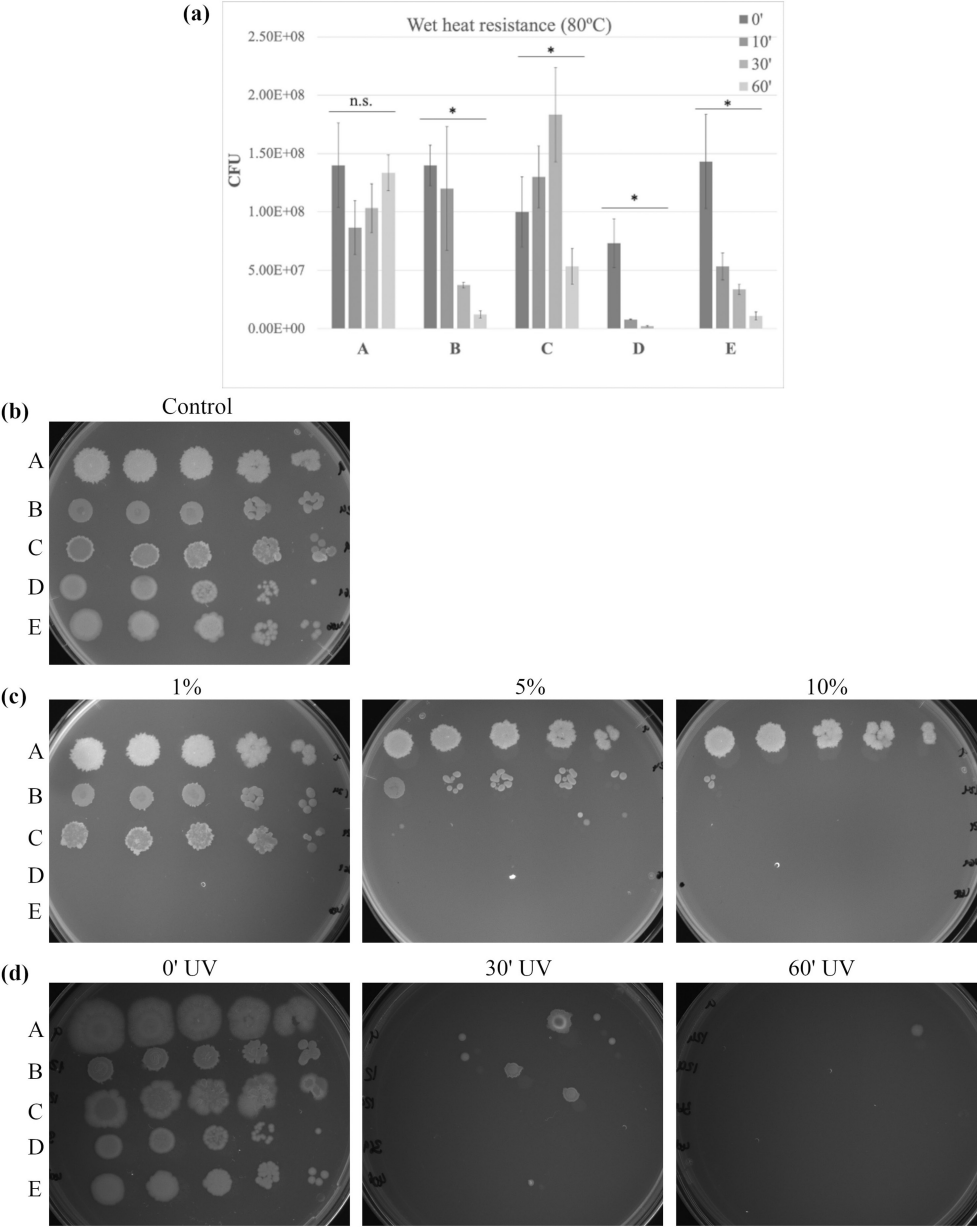
Spore resistance properties. Spores were exposed to indicated conditions, serial dilutions were plated on LB agar plates and assessed after overnight incubation. (a) Bacterial growth expressed as colony forming units (CFU) after exposure to wet heat at 80°C for 0 min (0′), 10 min (10′), 30 min (30′) and 60 min (60′). (*) indicates significant difference (*p* < 0.05); (n.s.) indicates no significant difference as detected by one‐way‐ANOVA test. (b) Bacterial growth of untreated samples (Control). (c) Bacterial growth after treatment with oxidising agents at different concentrations (1%, 5% and 10% bleach). (d) Bacterial survival after exposure to ultraviolet radiation for 0 min (0′UV), 30 min (30′UV) and 60 min (60′UV). Dilution factors on images (b–d) from left to right: 10^−1^, 10^−2^, 10^−3^, 10^−4^, 10^−5^. (A) UJA_LIN_009, (B) UJA_LIN_124, (C) UJA_LIN_129, (D) UJA_MA_369 and (E) UJA_MA_406.

We next investigated the resistance of isolate spores to wet heat, oxidising agents (household bleach) and ultraviolet radiation (Figure 3a–d). All strains were sensitive to UV treatment losing viability after ultraviolet radiation exposure > 30 min (Figure 3d). Spores of the UJA_LIN_009 strain demonstrated the highest resistance of all tested isolates, as it was only sensitive to UV treatment > 30 min but resistant to other treatments. Spores of the strain UJA_LIN_124 demonstrated progressive reduction of viability after increasing wet heat exposure at 80°C (0–60 min) as well as decreasing colony formation ability after oxidising agent treatment. Notably, at 10% bleach concentration, strains almost completely lost viability.

Spores of the isolates UJA_MA_369 and UJA_MA_406 also demonstrated increasing sensitivity to prolonged wet heat exposure, with the former losing viability after incubation times longer than 30 min. Both strains lost viability after oxidising agent treatment already at low concentrations (1% bleach).

Finally, for spores of UJA_LIN_129, increased colony forming ability was detected for wet heat treatment up to 30 min and a drop in viability at 60 min. The increase in viability could presumably reflect spore heat activation for germination (Koopman et al. 2022). The spores furthermore demonstrated intermediate resistance towards bleach treatment as compared to other isolates, as they could tolerate oxidising agents > 1% but < 5%.

### 3.5 Olive Sporobiota Isolates Spore Morphology and Ultrastructure

**FIGURE 4:**
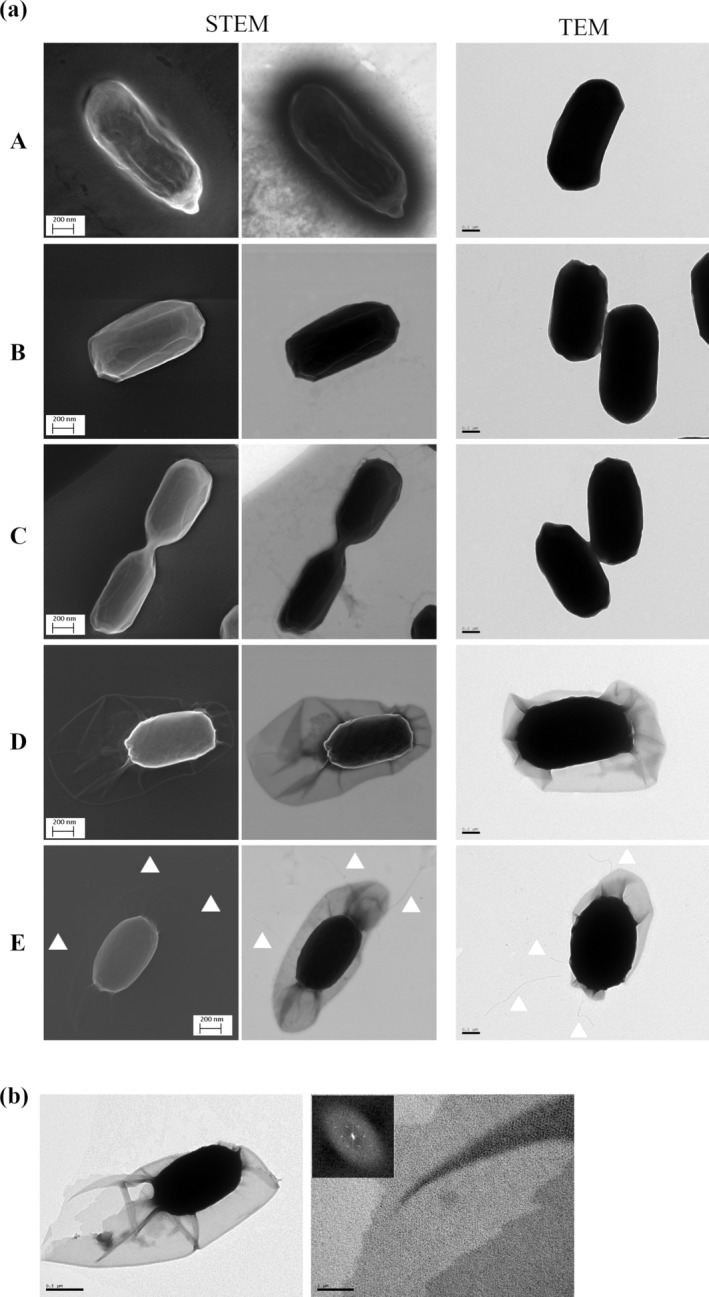
Spore ultrastructure as assessed by (a) Scanning transmission electron microscopy (STEM) and Transmission electron microscopy (TEM). (A) UJA_LIN_009, (B) UJA_LIN_124, (C) UJA_LIN_129, (D) UJA_MA_369 and (E) UJA_MA_406. (b) TEM electron micrograph of UJA_MA_369 spore (left) and its exosporium at higher magnification (right), including diffraction spots in Fourier transform (inlay). Sizes of scale bars are indicated.

Electron microscopy of negatively stained spores was used to investigate the ultrastructure of indigenous olive sporobiota isolates (Figure 4). Given that the exosporium is the first point of contact of the spore with its environment (Stewart 2015), particular attention was paid to the presence/absence of this layer. Both scanning (STEM) and transmission (TEM) electron micrographs revealed the expected bacillar shape of endospores. Moreover, UJA_LIN_009 (Figure 4a‐A) and UJA_LIN_124 (Figure 4a‐B) were surrounded by a tightly fitted outermost layer, presumably the spore coat. UJA_LIN_129 (Figure 4a‐C) also was devoid of an exosporium but surrounded by a tightly fitted coat structure.

Electron micrographs of UJA_MA_369 on the other hand clearly showed the presence of an outermost exosporium layer loosely surrounding the underlying spore integuments (Figure 4a‐D). The layer seemed asymmetrical, being closer to one spore pole than the other and appeared to have a hairy nap. Closer examination of transmission electron micrographs furthermore might hint towards the presence of a two‐dimensional crystal layer as it yielded a diffraction pattern in the form of diffraction spots in Fourier transforms (Figure 4b). These findings suggest the presence of a crystalline basal layer in the isolates' exosporium structure.

Finally, electron micrographs of UJA_MA_406 also demonstrated the presence of a loosely fitted exosporium surrounding the spore. Unlike the UJA_MA_369 spore ultrastructure, this outermost layer showed a more symmetrical distribution around the underlying integuments. In addition, several filamentous extensions of different lengths were visibly extending from the exosporium surface (Figure 4a‐E). These appendices branched out at the tips into several threads.

### 3.6 Genomic Analysis of UJA_MA_369

#### 3.6.1 Genome Assembly and Analysis

**FIGURE 5:**
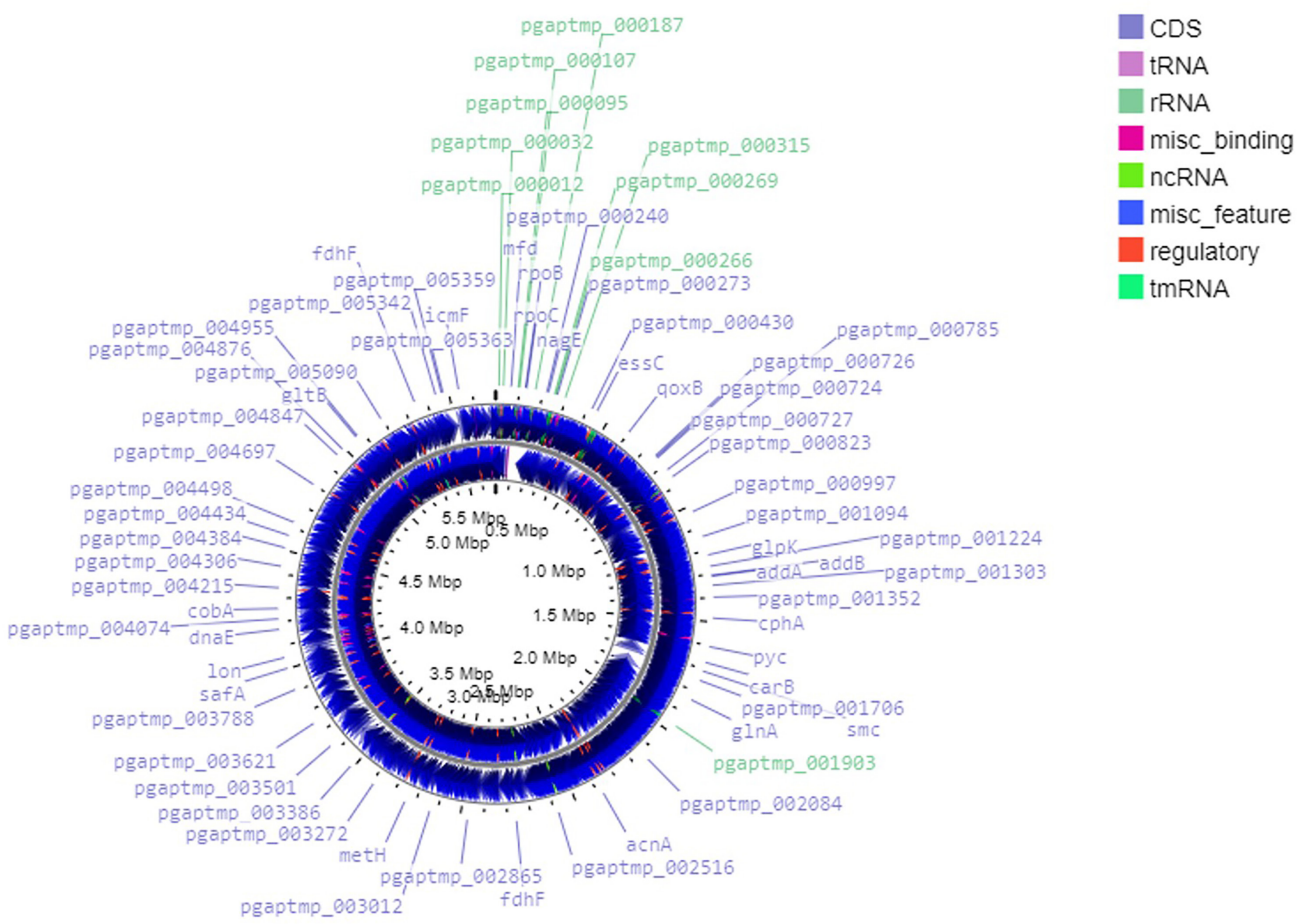
Circular map of the UJA_MA_369 chromosome. Coding DNA Sequences (CDS) elements, tRNA, rRNA, ncRNA, tmRNA and other regions are indicated.

Given the antimicrobial potential of isolate UJA_MA_369 as determined in our previous studies (Manetsberger, Caballero Gómez, Benomar, et al. 2023; Manetsberger et al. 2024), as well as promising results obtained in this study regarding resistance/sensitivity towards spore treatment, this olive sporobiota candidate was selected for a more in‐depth assessment through whole genome sequencing. First, average nucleotide identity (ANI) for taxonomic identification against the type strain with the highest z‐score value showed the isolate to be closely related to *Peribacillus frigoritolerans* FJAT‐2396 (ANIb = 96.73%, 83.74% of sequence aligned). Its genome consists of a single circular chromosome of 5,698,984 bp with an estimated 40.4% G + C mol content (Figure 5). ORF annotation predicted 5.450 ORFs, including 88 tRNA genes (1.6% of all genes) and 42 rRNA genes (0.77% of all genes).

#### 3.6.2 Genomic Islands

**FIGURE 6:**
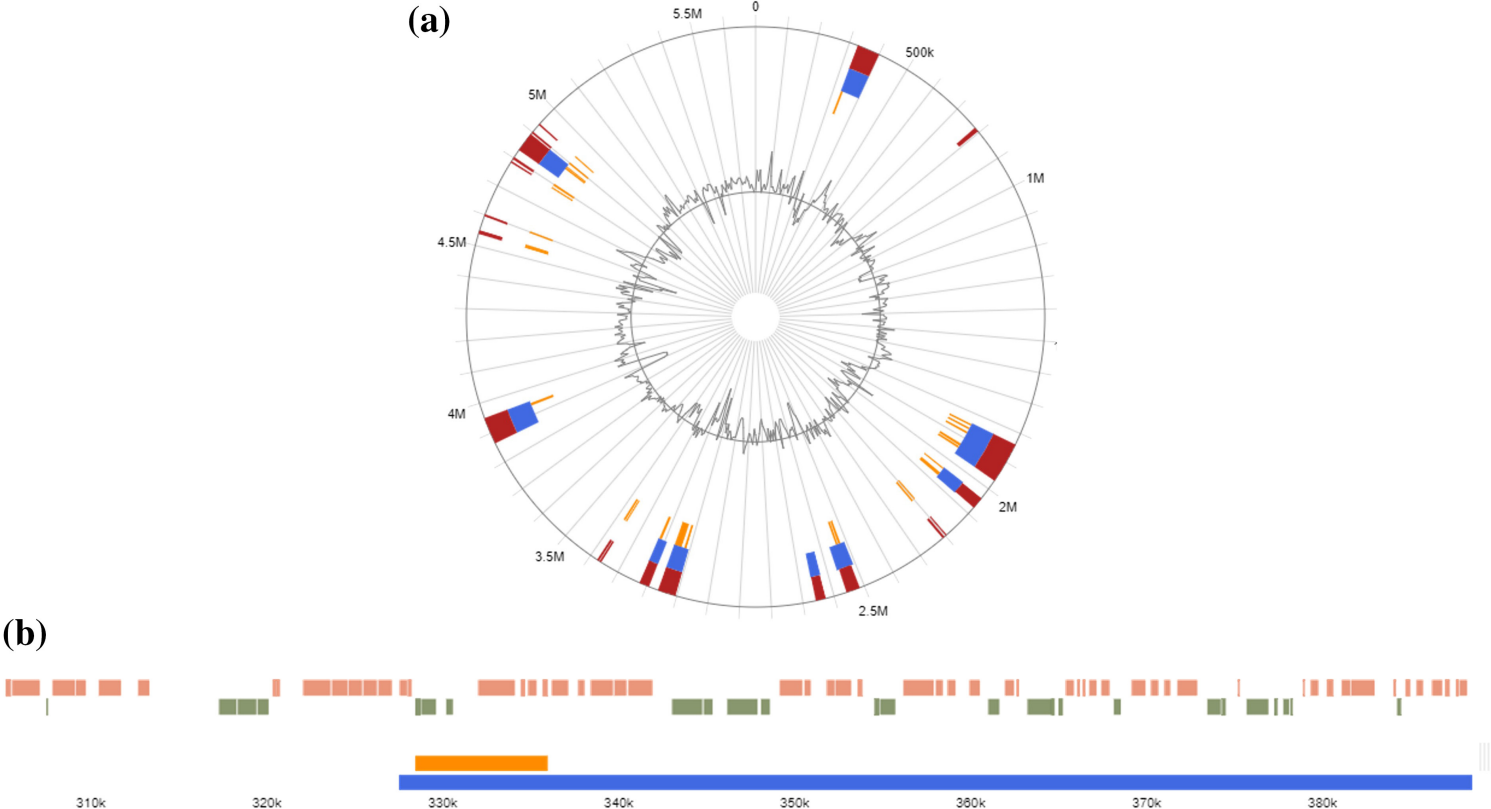
Prediction of genomic islands in UJA_MA_369. Circular (a) and linear (b, individual item in the circular view) visualisation of predicted genomic islands are shown, with blocks coloured according to the prediction method (Bertelli et al. 2017): IslandPick (green), IslandPath‐DIMOB (blue) and SIGI‐HMM (orange).

Bioinformatic analysis furthermore indicated the presence of several genomic islands (GI) in the UJA_MA_369 genome (Figure 6). A total of 15 GI were predicted with a length of 8455, 14,378, 106,212, 34,675, 16,605, 25,285, 31,336, 55,481, 29,829, 15,120, 77,559, 12,004, 6680, 19,688 and 105,217 bp (Tables 3 and S1). No prophage associated genes were detected, but GI_8 contained several anti‐phage associated elements.

**TABLE 3:** Summary of genetic islands detected in the UJA_MA_369 genome.

Genomic Island (GI)	Start	End	Length (bp)	No. of predicted genes
GI_1	327,896	336,351	8455	83
GI_2	780,938	795,316	14,378	14
GI_3	1,837,717	1,943,929	106,212	162
GI_4	2,037,030	2,071,705	34,675	63
GI_5	2,194,612	2,211,217	16,605	11
GI_6	2,515,558	2,540,843	25,285	93
GI_7	2,627,427	2,658,763	31,336	30
GI_8	3,104,467	3,159,948	55,481	83
GI_9	3,192,542	3,222,371	29,829	37
GI_10	3,360,470	3,375,590	15,120	14
GI_11	3,865,949	3,943,508	77,559	82
GI_12	4,538,065	4,550,069	12,004	15
GI_13	4,595,740	4,602,420	6680	9
GI_14	4,783,477	4,803,165	19,688	15
GI_15	4,829,557	4,934,774	105,217	88

#### 3.6.3 Bacteriocin Production

**FIGURE 7:**
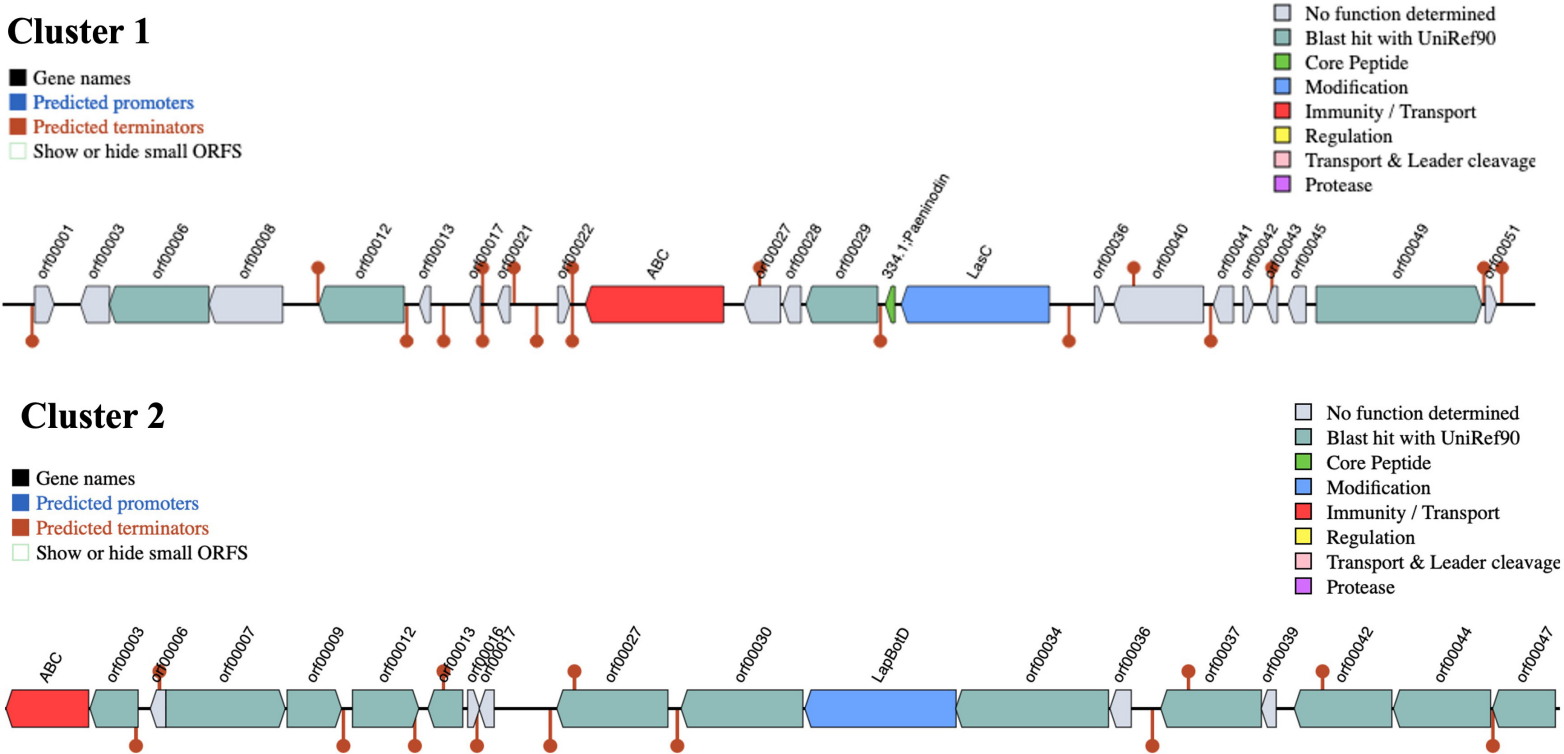
Bacteriocin Clusters 1 and 2 as predicted by BAGEL4.

Regarding the production of bioactive compounds, bioinformatic analysis yielded the presence of two genomic clusters with putative antimicrobial activity (Figure 7). The first cluster encodes 23 genes, some of which might be involved in antimicrobial activity, including homologues of a Lipid A export ATP‐binding/permease protein MsbA (ABC) and a LasC aparagine synthetase. Most importantly, a bioactive core peptide was predicted, showing high similarity to the lasso peptide paeninodin. The second cluster contains 19 ORFs, including homologues of the spermidine/putrescine import ATP‐binding protein PotA (ABC) and molybdate/tungstate transport system permease protein WtpB (orf00003). The cluster furthermore contains homologues of the trifolitoxin‐processing protein TfxB (orf00030) involved in trifolitoxin biosynthesis as well as uncharacterised protein in tfuA 3′region (LapBotD) putatively involved in azoline formation. No biogenic amine production‐associated protein sequences were detected in the UJA_MA_369 genome.

#### 3.6.4 Evaluation of UJA_MA_369 Safety Properties

Regarding the assessment of its safety properties, in silico prediction indicated the presence of two virulence factors in the UJA_MA_369 genome: VFG000682 (gb|AAF13663) and VFG016400 (gb|WP_000502776). Both virulence factor genes (VFG) are predicted to be involved in the biosynthesis of the bacterial capsule, with VFG000682 (gb|AAF13663) identified as a homologue of the *Bacillus anthracis* capsule biosynthesis protein CapB and VFG016400 (gb|WP_000502776) a homologue of the *B. cereus* aminotransferase family protein polysaccharide capsule. CARD analysis did not detect any antibiotic resistance genes (ARG) in the UJA_MA_369 genome under the chosen settings.

## 4 Discussion and Conclusions

The current study examined genomic and biotechnological features of five selected isolates of the culturable olive sporobiota. The strains were the focus of in‐depth analysis given their promising antimicrobial activity against detrimental plant pests (e.g., *X. fastidiosa* ), making them promising candidates for their development into commercial bioactive compounds (Manetsberger, Caballero Gómez, Benomar, et al. 2023; Manetsberger et al. 2024).

Previous studies have demonstrated *Bacillus* spp.'s antimicrobial activity through the secretion of hydrolytic enzymes as well as biosurfactants into the culture medium which can be a powerful tool against plant pathogens (Ajuna et al. 2023). It is thus not too surprising that all isolates produced hydrolytic enzymes, and in some cases, biosurfactants. The latter notably also could have an implication in the strain's antimicrobial activity. As an example, the pathogen *X. fastidiosa* establishes itself in the xylem, forming stable biofilms, thus obstructing the plants conducive system and depriving the host of water and nutrients (EFSA Panel on Plant Health 2016; Sicard et al. 2018). Biosurfactants have been shown to act as biodispersants as well as biocides (Jimoh et al. 2023), and hence, it is conceivable that the secretion of hydrolytic enzymes and biosurfactants by olive sporobiota isolates could cause either the delay or complete disruption of such biofilms. This would effectively protect the plant, albeit potentially not eliminate the phytopathogen infection, rendering it asymptotic (which is in fact observed under field conditions). This hypothesis is particularly interesting for endophytic isolates UJA_LIN_124 and UJA_LIN_129, showing biosurfactant production as well as sharing a biological niche with *X. fastidiosa* in the plant/leaf interior. These findings are supported by the presence of surfactant‐producing genes in both isolates (Manetsberger et al. 2024).

The strains further showed interesting resistance properties both in vegetative and spore form. Good tolerance to high saline conditions (this study) in combination with resistance to heavy metals, antibiotics and fertilisers treatment, as shown by our previous studies (Manetsberger, Caballero Gómez, Benomar, et al. 2023), underline their excellent suitability as agricultural agents. On the other hand, the limited resistance to ultraviolet radiation could present a slight drawback, especially considering the high and extended sunlight exposure in arid climates typical for olive agriculture in the Mediterranean basin.

Regarding the bacteria ultrastructure, electron microscopy studies of intact negatively stained spores revealed the presence of a loosely attached exosporium on two of the olive sporobiota isolates. As a member of the *B. cereus* clade, perhaps this structure was to be expected in isolate UJA_MA_406, previously identified as *Bacillus mycoides* strain. Nonetheless, to our knowledge, this is the first report of such a structure in the species. Our studies have further revealed the presence of large pili covering the spore surface and extending out into several individual filaments at the far end, potentially indicating that the filaments are composed of several fibres as previously shown for members of *B. cereus* group species (Zegeye et al. 2021).

Unlike the underlying spore integuments, which show a good conservation, the exosporium has great ultrastructural variations, such as the presence of a full or reduced hairy nap (Manetsberger, Hall, and Christie 2015) or large appendages (Klavenes et al. 2002). These outermost surface features have been suggested to play a role in the mechanical interaction of the spore with its immediate environment, such as surface adherence, spore aggregation, biofilm formation as well as virulence (Zegeye et al. 2021). For environmental isolates, it is likely that the large extensions could be used to anchor the spores into the plant rhizosphere. An exosporium structure was also detected in a second isolate—UJA_MA_369 (previously assigned to the *Peribacillus* clade). To our knowledge, this is also the first report on the presence of an exosporium in *Peribacillus* as well as an underlying crystalline ultrastructure in this spore‐forming clade, similar to the one previously detected in the *B. cereus* family (Ball et al. 2008). However, further studies with more powerful electron microscopes are needed to confirm the crystalline nature of the UJA_MA_369 basal layer as well as determine its main structural components.

Although not obviously connected, those spores with an exosporium structure (UJA_MA_369 and UJA_MA_406) also exhibited a different growth morphology and biofilm formation than the other strains, that is, UJA_LIN_009, UJA_LIN_124 and UJA_LIN_129. The latter three notably formed floating biofilms at the air water interphase of stationary culture medium. High resolution imaging evidenced the presence of extended amorphous material into which the vegetative cells were embedded. This extensive biofilm formation is a common strategy also for other *Bacillus* spp., as by generating this microenvironment, the collective creates important advantages, such as making the bacteria (spores and cells) more resilient towards environmental challenges, ensuring their persistence in the complex soil matrix and providing higher resistance to antimicrobial components (Arnaouteli et al. 2021; Fessia et al. 2022; Morikawa et al. 2006). The fact that strains with an exosporium did not show pellicle formation could potentially suggest that both structures (exosporium and biofilm matrix) yet share some common functions, such as facilitating bacteria/host interaction (Arnaouteli et al. 2021; Stewart 2015). Moreover, it is interesting to note that two of the strains (UJA_LIN_124 and UJA_LIN_129) are of endophytic nature and were isolated from the interior of olive leaves (Manetsberger, Caballero Gómez, Benomar, et al. 2023). These strains both created strong floating biofilms, and it could by hypothesised that the extensive matrix formation is a strategy of the bacterium to populate the plant conducive system, such as the xylem. Future work should notably identify the composition of the matrix, for example, exopolysaccharides similar to *B. subtilis* biofilms (Arnaouteli et al. 2021), as well as extend genomic data on the presence of biofilm‐formation associated genes.

Whole genome sequencing (WGS) of the olive sporobiota isolate UJA_MA_369 allowed us to gain a deeper understanding of its antimicrobial properties and security. The strain was chosen based on its promising antimicrobial and biotechnological characteristics determined in the present and previous studies (Manetsberger, Caballero Gómez, Benomar, et al. 2023; Manetsberger et al. 2024). Taxonomic identification showed that UJA_MA_369 shared 96.73% sequence identity (83.74% aligned) with the type strain *P. frigoritolerans* FJAT‐2396. Interestingly, previous analysis using MALDI‐TOF identified the isolate as *P. simplex* strain (Manetsberger et al. 2024). That said, it has been shown that MALDI‐TOF identification may be error prone in some genus, for example, *Neisseria* (Hong, Bakhalek, and Taha 2019). This was partially attributed to frequent horizontal gene transfer which is also common in the genus *Bacillus* (Böhm et al. 2015); hence, variations in the two detection methods—although rare in nature—could be feasible.

*Bacillus* (and related) genus are known for their ability to produce a large variety of antimicrobial compounds, including antimicrobial peptides, for example, bacteriocins, lassopeptides and lantipeptides (Abriouel et al. 2011; Sumi et al. 2015). In this regard, the UJA_MA_369 genome also contained two gene clusters with predicted bacteriocin activity. The most interesting finding here was the identification of the bacteriocidal lasso peptide paeninodin, originally identified in the firmicute *Paenibacillus dendritiformis* C454 (Zhu et al. 2016). Lasso peptides have been shown to have a high thermal and proteolytic resistance as well as a high variety of biological activities, such as antimicrobial activity, or act as carriers for other active peptides (Cheng and Hua 2020). A second gene cluster of interest included genes putatively involved in trifolitoxin biosynthesis and azoline formation. The latter is active in the synthesis of linear azol(in)e‐containing peptides (LAPs), which often exhibit strong antimicrobial activity, including in plants, and have thus been previously proposed as biocontrol agents (Travin et al. 2019). Trifolitoxin is a potent antibiotic peptide which can increase plant competitiveness (Lethbridge et al. 2022; Robleto et al. 1998). It is thus possible that the active compound conferring antimicrobial activity to UJA_MA_369 is provided by one of the above mentioned compounds or a combination thereof. It can however not be excluded at this point that also other active peptides—with known or unknown sequences—are at least partially responsible for the antimicrobial activity of UJA_MA_369. Our next steps will consequently focus on the identification and characterisation of the detected and other antimicrobial metabolites, including polyketides and non‐ribosomally synthesised peptides.

Overall, it will be interesting in future studies to identify genomic features of the remaining four olive sporobiota isolates and provide a comparison of all five strains. Particular attention will also be paid to assessing biofilm biosynthesis, formation and stability. The greatest challenge and task will then be to adapt these promising microorganisms for their use as bioactive agents fit for modern agriculture.

## Author Contributions

**Julia Manetsberger:** conceptualization, investigation, funding acquisition, writing – original draft, writing – review and editing, methodology, supervision, data curation. **Natacha Caballero Gómez:** investigation, writing – review and editing. **Nabil Benomar:** conceptualization, writing – review and editing, supervision, investigation. **Graham Christie:** conceptualization, investigation, writing – review and editing. **Hikmate Abriouel:** conceptualization, investigation, funding acquisition, writing – original draft, writing – review and editing, supervision.

## Conflicts of Interest

The authors declare no conflicts of interest.

## Supporting information


**Figure S1.** Screening of biosurfactant production of (A) UJA_LIN_009, (B) UJA_LIN_124, (C) UJA_LIN_129, (D) UJA_MA_369 and (E) UJA_MA_406 in comparison to sterile water as negative control (H_2_O) and a 10% SDS solution as positive control (SDS).

**Table S1.** Prediction of genomic islands in the UJA_MA_369 genome and hypothetical gene annotations.

## Data Availability

The data that support the findings of this study are openly available in the European Nucleotide Archive (accession number PRJEB80151).
